# Manipulating the duration of picoinjection controls the injected volume of individual droplets

**DOI:** 10.1063/5.0206830

**Published:** 2024-07-02

**Authors:** R. Thakur, D. Weitz

**Affiliations:** 1 Harvard-Massachusetts Institute of Technology Division of Health Sciences and Technology, Massachusetts Institute of Technology, Cambridge, Massachusetts 02139, USA; 2 John A. Paulson School of Engineering and Applied Sciences, Harvard University, Cambridge, Massachusetts 02138, USA; 3 Department of Physics, Harvard University, Cambridge, Massachusetts 02138, USA and Wyss Institute for Biologically Inspired Engineering, Harvard University, Cambridge, Massachusetts 02115, USA

## Abstract

The ability to add reagents into droplets is required in many microfluidic workflows. Picoinjection can address this need; however, it is unable to control the injection volume for each individual droplet. Here, we present an improved picoinjection method that can inject controlled volumes into individual droplets. We achieve this by adjusting the injection duration for each picoinjection event. This improved picoinjection method can be used to create complex microfluidic workflows that are able to control the biochemical composition of individual droplets.

## I. INTRODUCTION

Droplet microfluidics has transformed biological research by miniaturizing molecular biology assays. Droplets serve as picoliter bioreactors replacing conventional milliliter test tubes. With this miniaturization, droplet microfluidic workflows dramatically reduce reagent consumption.^1^ Moreover, because microfluidic droplets can be generated and sorted at kilohertz rates, these workflows also have incredibly high throughputs.^2–6^ Together, these two features enable droplet microfluidic workflows to process hundreds of thousands of independent biological reactions in minutes. However, to truly replicate conventional molecular biology assays in a miniaturized droplet format, microfluidic technologies must also be capable of adding reagents to each bioreactor following droplet formation. Adding reagents into a droplet closely mirrors the pipetting of reagents into a test tube as required in many workflows. Although seemingly simple, this operation is challenging to translate to a microfluidic format; the interface of the droplet must be temporarily disrupted to add the reagents and then restored to prevent cross-contamination between droplets. Microfluidic picoinjection can address this need by temporarily electro-coalescing the interface between a droplet and the picoinjector, a channel carrying the desired reagents.^7–12^ When the interface is disrupted, the pressure difference between the picoinjector and the droplet delivers a precise volume of fluid into the droplet. The volume injected can be controlled by specifying the pressure applied to the picoinjector and the velocity of the flowing droplets. These parameters, however, cannot be adjusted rapidly enough to control the injection volume for each individual droplet.^8^ A picoinjection method capable of controlling the injection volume for each droplet would enable tailoring each droplet's composition. With this capability, microfluidic workflows could picoinject different reagent volumes according to a biological reporter or incorporate real time feedback to improve the uniformity of picoinjection. This improved picoinjection method would enable microfluidic workflows to replicate arbitrary pipetting operations in a miniaturized and autonomous device.

In this paper, we present a picoinjection method that can control the injection volume for each droplet. We achieve this by controlling when picoinjection is initiated relative to the droplet position in the flowing channel. In our approach, we detect each droplet and initiate picoinjection by electro-coalescing the picoinjector–droplet interface after a portion of the droplet has crossed the picoinjector nozzle. By initiating picoinjection after this controlled delay, we lower the effective injection duration thereby decreasing the injection volume. We can vary this delay from zero to the total transit time of the droplet across the picoinjector. This establishes a wide dynamic range of injection volumes that can be adjusted with picoliter precision. Importantly, our approach can control the injection volume for each droplet because this delay can be adjusted as rapidly as the droplets can be picoinjected.

## II. MATERIALS AND METHODS

We fabricate a microfluidic picoinjection device with 40-*μ*m-tall channel walls using soft lithography.^13^ Our device geometry includes an inlet to inject premade 70-*μ*m-diameter water-in-oil droplets and an oil co-flow to space the droplets before they reach the picoinjector. This spacer oil and the continuous phase of the emulsion are both Novec HFE-7500 with 1% biocompatible surfactant.^14^ We use 100** ***μ*M fluorescein sodium salt dissolved in phosphate buffered saline as the aqueous phase of the emulsion. We include fluorescein dye in the aqueous phase to make each droplet fluorescent such that we can detect them as they flow in the channel. We inject the droplets into the picoinjection device using a vertically oriented syringe pump. Due to their buoyancy, the droplets pack at the top of the syringe and enter the device at a high-volume fraction with minimal excess oil. We introduce the droplets at a flow rate of 100** ***μ*l/h and introduce the spacer oil at a flow rate of 500** ***μ*l/h. At these flow rates, the picoinjection device has a processing throughput of 150 droplets per second.

We pressurize the fluid in the picoinjector to 150 mBar such that the oil–water interface is pinned in the 5 *μ*m nozzle of the picoinjector. The pinned water–oil interface creates a Laplace pressure that balances the pressure differential between the fluid in the picoinjector and the flowing oil channel.^7^ In this configuration, a thin oil film separates the picoinjection fluid and the flowing droplets.^7,8^ We initiate picoinjection by coalescing this oil film with an electrical signal applied directly to the fluid in the picoinjector. We chose to apply the electric field directly to the picoinjection fluid to avoid the fabrication of embedded electrodes.^8^ For each picoinjection event, we generate a 40 kHz sine wave pulse with a duration of 100 *μ*s. This electro-coalescence signal briefly disrupts the picoinjector–droplet interface and allows fluid to flow from the picoinjector into the droplet.^7,8^

**FIG. 1.**
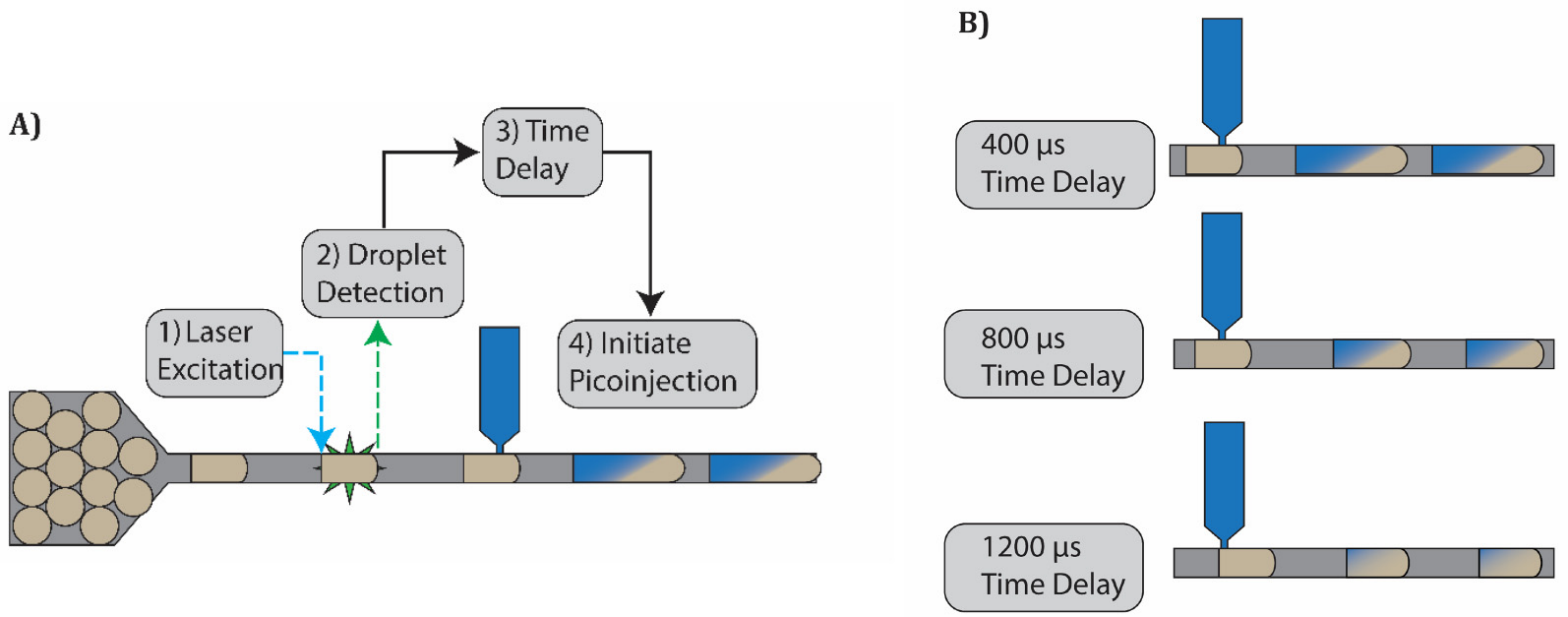
Schematic of picoinjection with delayed initiation. We control the injection duration by controlling when picoinjection is initiated. (a) We detect each droplet by measuring its fluorescence as it flows across a laser focused upstream of the picoinjector. Following a prescribed delay, the electric field is momentarily turned on to initiate picoinjection. (b) The injected volume varies with the delay time.

We determine the moment the droplets arrive at the picoinjector nozzle and control when we apply the electro-coalescence signal relative to this arrival time. We measure each droplet's emission signal as it crosses a 488 nm excitation laser focused upstream of the picoinjector. Given the laser's position and the velocity of the droplets, we can determine when each droplet arrives at the picoinjector nozzle. Using custom Labview software, we specify when the electro-coalescence signal is applied relative to the moment the droplet arrives at the picoinjector. By delaying the application of this electro-coalescence signal, we can shorten the injection duration and therefore decrease the injection volume as shown in Fig. 1. We record videos of each picoinjection event using a high-speed camera. To synchronize camera recordings with picoinjection, we send a signal to the trigger pin of the camera when each droplet is detected.

## III. RESULTS AND DISCUSSIONS

**FIG. 2.**
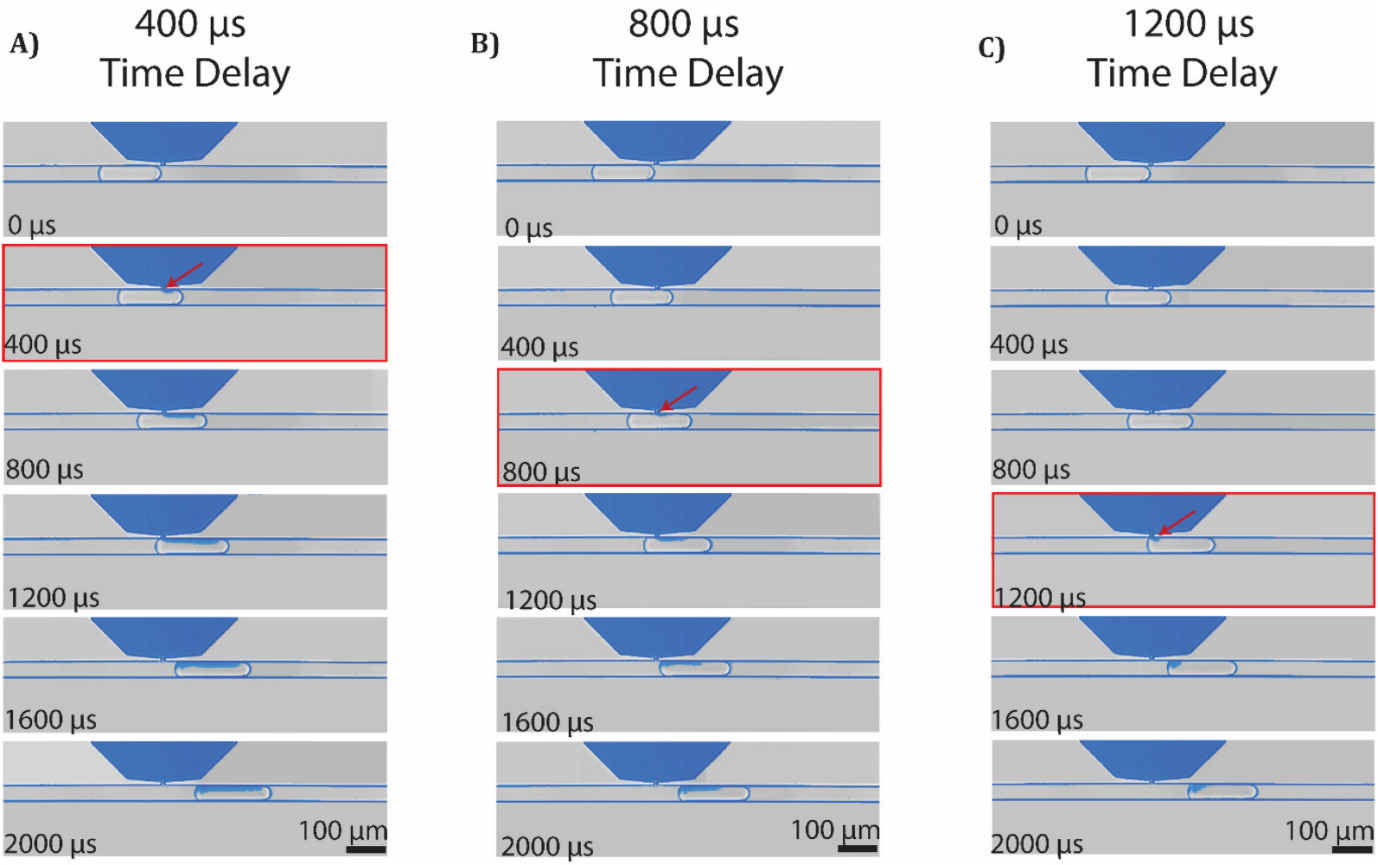
Picoinjection is initiated at the exact time at which we apply the electro-coalescence signal at (a) 400, (b) 800, and (c) 1200 *μ*s following the droplet's arrival at the picoinjector. The images with red borders indicate the frame corresponding to initiation of picoinjection. Red arrows indicate fluid from the picoinjector beginning to flow into the droplet.

To demonstrate that we can precisely control the initiation of picoinjection, we vary the exact time at which we apply the electro-coalescence signal and record videos of each picoinjection event. To visualize the moment picoinjection is initiated, we use 1% calligraphy ink in phosphate buffered saline as the fluid for picoinjection. The difference in absorbance between the fluid in the picoinjector and the droplet allows us to identify when the picoinjector–droplet interface is disrupted and when fluid begins to flow into the droplet. Upon detection of each droplet, we apply a 200 V electro-coalescence signal to the picoinjector incorporating a software specified delay. By varying the length of this delay, we control the time at which picoinjection is initiated relative to the moving droplet. Then, we verify that the picoinjector–droplet interface is disrupted in the video frame corresponding to the specified delay as shown in Fig. 2. This demonstrates that picoinjection can be initiated at the precise timing specified by our control software.

**FIG. 3.**
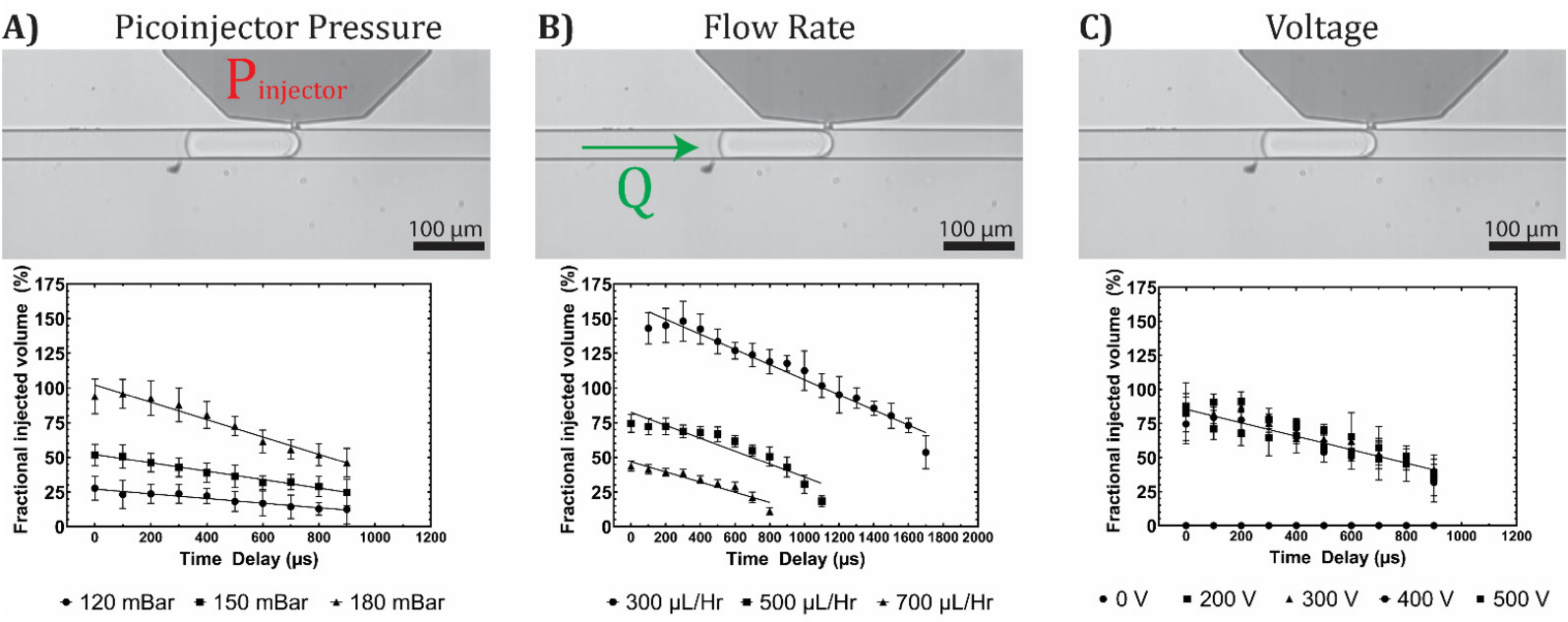
The injected volume can be controlled by adjusting the picoinjector pressure, droplet flow rate, and the injection duration. The volume of fluid injected into the droplet varies linearly with the injection duration. (a) Graph shows the fractional injected volume for picoinjector pressure at 120, 150, and 180 mBar. By increasing the picoinjector pressure, the amount of injected fluid per unit time increases. (b) Graph shows the fractional injected volume for oil spacer flow rates at 300, 500, and 700 *μ*l/h. By increasing the oil spacer flow rate, the total injection duration decreases. (c) Graph shows the fractional injected volume for picoinjector voltages at 0, 200, 300, 400, and 500 V. If the applied voltage is sufficient to induce coalescence of the injector–droplet interface, the injected fractional volume is constant with increasing voltage. The error bars correspond to the standard deviation in the fractional injected volume, as measured for 300 picoinjection events.

We quantify the amount of fluid injected by measuring the change in droplet volume after picoinjection. When compressed in the channel, the droplets are shaped approximately like rounded cylinders.^8^ Therefore, the droplet volume is linearly related to the droplet length in the channel. For each picoinjection event, we extract a video frame before and after picoinjection. We measure the distance between the leading and trailing edge of each droplet and record the change in droplet length. To calculate the fractional injected volume, we normalize the change in droplet length to the original length of the droplet before injection. By varying the time delay between droplet detection and the initiation of picoinjection, we control the injection duration and quantify the fractional injected volume. The fractional injected volume decreases linearly with this time delay as shown in Fig. 3. In addition, we vary the picoinjector pressure, oil flow rate, and picoinjector voltage to characterize how these parameters affect the fractional injected volume. Increasing the picoinjector pressure increases the injected volume per unit time. This is reflected in the increased slope of the injected volume's dependence on time delay, as shown in Fig. 3(a). Decreasing the oil flow rate increases the transit time of the droplet across the picoinjector and, therefore, increases the total injection duration. This results in a larger injected volume at the same delay settings as shown by the vertical shift in the injected volume in Fig. 3(b). In these experiments, we observe no change in the injected volume when we vary the picoinjector voltage, as shown in Fig. 3(c).

**FIG. 4.**
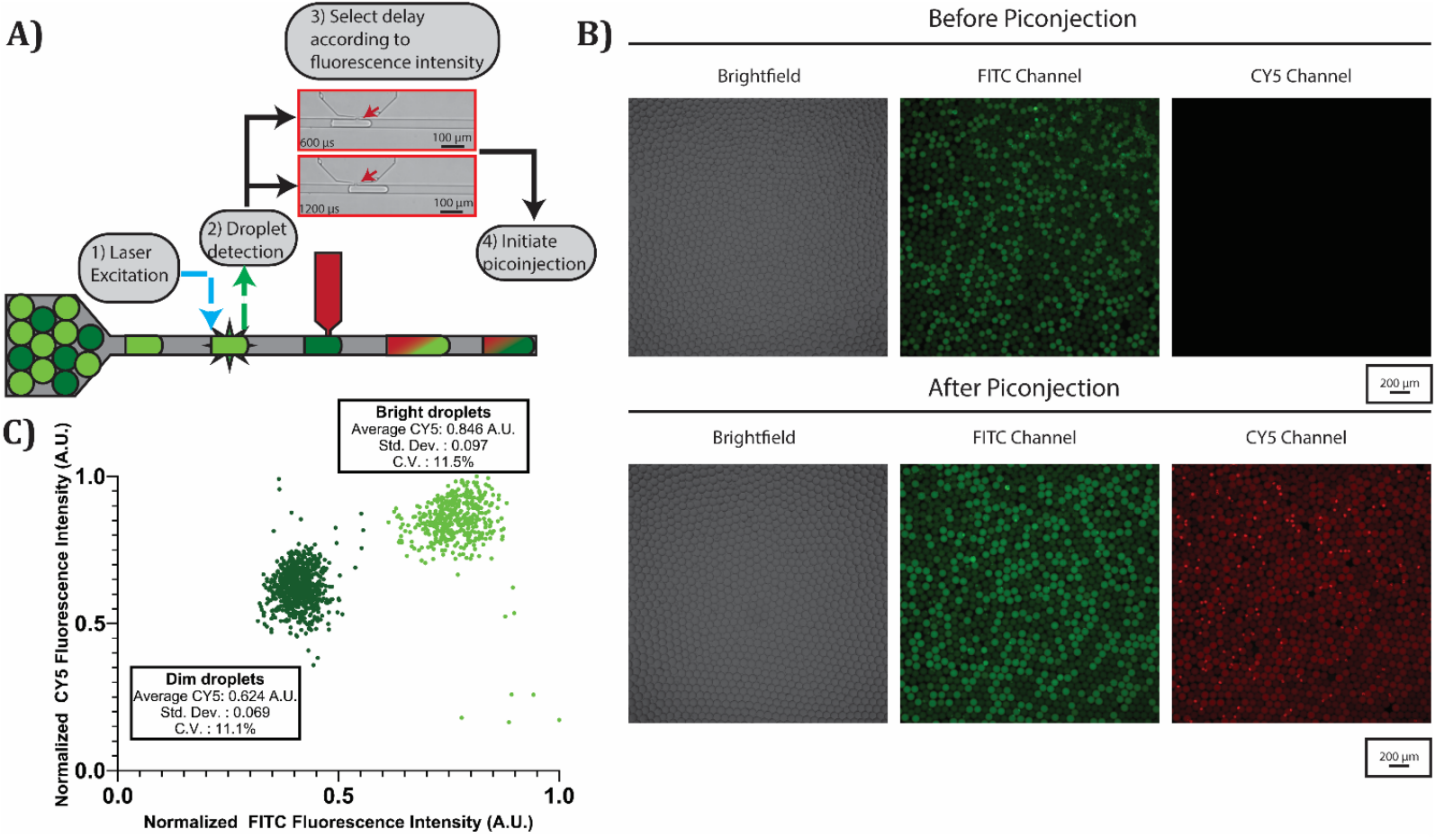
The injected volume can be controlled on an individual droplet basis. (a) A mixed emulsion of bright and dim fluorescein droplets is injected into the picoinjection device, and the fluorescence intensity of each droplet is measured. Picoinjection is then initiated after a 600 *μ*s delay for bright droplets and a 1200 *μ*s delay for dim droplets. Sulfo-cyanine 5 is included in the picoinjector to visualize the injected volume. (b) Microscope images of droplets before and after picoinjection. (c) Scatterplot showing the amount of sulfo-cyanine 5 injected into the bright droplet subpopulation and the dim droplet subpopulation. Each data point represents the normalized fluorescence intensity in the FITC and CY5 channel of a droplet following picoinjection. 1124 droplets were analyzed.

To demonstrate that this picoinjection method can control the composition of individual droplets, we inject unique volumes according to each droplet's fluorescence intensity. We prepare an emulsion composed of bright and dim fluorescent water-in-oil droplets, as shown in Fig. 4(b), top. The bright and dim droplets contain 75 and 25** ***μ*M fluroscein sodium salt, respectively. We inject this emulsion into our picoinjection device and detect the fluorescence intensity of each droplet. Then, we initiate picoinjection after a 600 *μ*s delay for the bright droplets and a 1200 *μ*s delay for the dim droplets, as shown in Fig. 4(a). By specifiying different delays, we control the injection duration and, therefore, the injection volume for each droplet subpopulation. With these delay settings, our picoinjection device should inject a larger volume into the bright droplets. To visualize the volume injected into each droplet, we use 100** ***μ*M sulfo-cyanine-5 in phosphate buffered saline as the fluid in the picoinjector. We image the droplets following picoinjection and see a bimodal fluorescence intensity distribution in the cyanine-5 channel, as shown in Fig. 4(b). This bimodal fluorescence intensity distribution indicates that our device injected two different fractional volumes as expected. To quantify the volume injected into each subpopulation, we plot each droplet's normalized fluorescence intensity and label it as a bright or dim droplet according to its fluorescence intensity in the fluroscein channel, as shown in Fig. 4(c). The bright droplet subpopulation on average had a higher fluorescence intensity in the cyanine-5 channel, indicating a larger injected volume. Both the dim and bright droplet subpopulations had approximately a 11% coefficient of variation in the cyanine-5 fluorescence channel. This variablity in the injected volume can arise from imprecision in the pressure controller and the syringe pump, as well unstable pinning of the oil–water interface in the picoinjector nozzle. Because this picoinjection method controls the injected volume by manipulating the injection duration, variations in the input droplet size can lead to additional variation in the injected volume. A larger droplet experiences a longer transit time across the picoinjector and, therefore, will receive a larger injected volume with the same delay settings. This additional variability can be minimized in the future by programming the control software to modulate the delay according to the droplet's detected length. For both droplet subpopulations, there are some outliers in the plotted CY5 intensities, as shown in Fig. 4(c). We attribute these outliers to our picoinjection control software miscategorizing the droplet. Our control software measures the fluorescence intensity and size of each droplet and specifies the injection duration according to the droplet's assigned droplet subpopulation. Some droplets were likely mislabeled as bright or dim droplets and received the incorrect injection duration. Additionally, some droplets’ detected fluorescence intensity was brighter than the upper threshold in our picoinjection software. As a result, these droplets were not assigned to either subpopulation and were not injected, as shown by the points in the bottom right corner of Fig. 4(c). Despite these outliers, this fluorescence image data validates that our improved picoinjection method can control the injection volume for each individual droplet from a heterogeneous sample.

## IV. CONCLUSIONS

We have developed an improved picoinjection method that can control the injection volume for individual droplets. We control the injection volume by adjusting the injection duration. Because we can adjust the injection duration as rapidly as we can detect droplets, we can control the injection volume for each picoinjection event. For our method, the range of injection volumes is tuned by initially adjusting the picoinjector pressure and oil flow rate, while the specific injection volume for each droplet is controlled by adjusting the injection duration. This enables fine tuning of the dynamic range of the picoinjector to match the needs of the overall microfluidic workflow. The extent that the injection duration can be varied by this method is dependent on the relative size of the droplets and picoinjector device height. The device height must be smaller than the droplet diameter such that the droplets enter as elongated cylinders. The length of the droplet in the channel and the transit time of the droplet determine the range of injection durations that can be selected. In our experiments, we did not see any adverse impact on droplet stability or polydispersity when the droplets were compressed in this configuration.

To adjust the injection duration accurately for each droplet, we need to identify the precise moment the droplet arrives at the picoinjector nozzle. In this paper, we accomplished this by fluorescently labeling each droplet and monitoring its fluorescence at a fixed position upstream of the picoinjector. In many applications of this method, this additional dye would not be necessary. Any existing fluorescent signal in the microfluidic workflow, such as a reporter for enzyme activity or a nucleic acid amplification probe, would generate a signal that could be used to detect the arrival of the droplet. It is also possible to detect the arrival of the droplet by measuring the side scatter generated from the traveling droplet's water–oil interface. This flexibility in detection modality makes this picoinjection method easy to incorporate into existing microfluidic workflows.

By incorporating our improved picoinjection method, microfluidic workflows can autonomously control the composition of individual droplets at high throughputs. This functionality can be leveraged to improve the precision of picoinjection and select unique injection volumes according to a biological reporter. Picoinjection is often performed on droplets that have been generated on a separate device. In these cases, the injected droplets may be polydisperse in size. Our approach could be used to detect the width of polydisperse droplets and control the injection duration such that the injection volume is uniform for all droplets. In directed evolution applications, picoinjection can be used to deliver a substrate to droplets containing unique enzyme variants. Our approach can be used to inject different amounts of a substrate according to a fluorescent reporter indicating the enzyme's expression level. By doing so, it is possible to quantify the reaction rate of an enzyme more accurately and screen for high performing variants. Our approach enables microfluidic workflows to tailor each droplet's content and should be useful in translating complex screening assays into a microfluidic format.

## Data Availability

The data that support the findings of this study are available within the article.
